# Tellurium as a high-performance elemental thermoelectric

**DOI:** 10.1038/ncomms10287

**Published:** 2016-01-11

**Authors:** Siqi Lin, Wen Li, Zhiwei Chen, Jiawen Shen, Binghui Ge, Yanzhong Pei

**Affiliations:** 1 Key Laboratory of Advanced Civil Engineering Materials of Ministry of Education, School of Materials Science and Engineering, Tongji University, 4800 Caoan Road, Shanghai 201804, China; 2 Beijing national laboratory for condensed matter physics, Institute of physics, Chinese academy of science, Beijing 100190, China

## Abstract

High-efficiency thermoelectric materials require a high conductivity. It is known that a large number of degenerate band valleys offers many conducting channels for improving the conductivity without detrimental effects on the other properties explicitly, and therefore, increases thermoelectric performance. In addition to the strategy of converging different bands, many semiconductors provide an inherent band nestification, equally enabling a large number of effective band valley degeneracy. Here we show as an example that a simple elemental semiconductor, tellurium, exhibits a high thermoelectric figure of merit of unity, not only demonstrating the concept but also filling up the high performance gap from 300 to 700 K for elemental thermoelectrics. The concept used here should be applicable in general for thermoelectrics with similar band features.

## Abstract (web summary)

Good thermoelectric materials are often complex compounds. Here, the authors reveal that elemental tellurium has a high thermoelectric figure of merit between 300 and 700 K when doped with As, with the potential advantages of easy preparation and relative isotropy.

## Graphical abstract

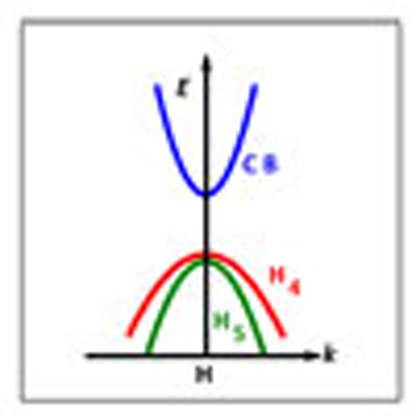

Thermoelectric devices, which enable a direct conversion between heat and electricity based on either Seebeck or Peltier effects, have attracted increasing interest as a sustainable and emission free solution to the imminent global energy crisis and environment pollution for a few decades^1^. The performance of a thermoelectric material is determined by the dimensionless figure of merit, *zT*=*S*^*2*^*σT*/(*κ*_E_+*κ*_L_), where *S*, *σ*, *κ*_E_, *κ*_L_, and *T* are the Seebeck coefficient, electrical conductivity, electronic thermal conductivity, lattice thermal conductivity and absolute temperature, respectively.

Because the electrical properties including *S*, *σ* and *κ*_E_ couple with each other strongly, a simple improvement in one of these three parameters usually leads to a compensation in the other two, resulting in the difficulty for enhancing *zT*. Minimizing the lattice thermal conductivity (*κ*_L_), the only one independent material property, has been proven to be effective through nanostructuring^2^,^3^,^4^,^5^,^6^, liquid phonons^7^,^8^ and lattice unharmonicity^9^,^10^ in the recent 15 years.

Alternatively, recent band engineering efforts aiming to obtain a high number of degenerated valleys (*N*_v_) (refs 11, 12, 13, 14, 15, 16, 17, 18), a low carrier inertial mass^19^ and a weak scattering^20^,^21^ has also led to great success in increasing the figure of merit *zT* (ref. 22). Taking the strategy of increasing *N*_v_ by converging two different valence (or conduction) bands in the k-space as an example, which has been well-demonstrated in *p*-type PbTe (ref. 15) and other IV–VI (ref. 23) semiconductors, *zT* has found to be increased significantly.

A straightforward understanding on how band convergence leading to high thermoelectric performance, is the increased conducting channels for high electrical conductivity, without affecting the Seebeck coefficient that is determined by the position of Fermi level and scattering mechanism^11^. Being slightly different from the band convergence where two or more band branches having similar energy but unnecessarily the same k-space location, nested bands have not only similar energy but also the same k-space location. In spite of the difference in k-space location between band convergence and nestification, the aligned bands in both cases should equally contribute to the transport of charge carriers, leading to a superior electrical performance for high thermoelectric efficiency.

Band nestification often occurs in well-known simple semiconductors such as group IV elements and III–V compounds, particularly in *p*-type conduction, this is mainly due to the splitting of degenerate bands by spin-orbit interaction^24^. This interesting band feature has led these materials to be playing important roles in the electronic industry for many decades, and many of these semiconductors have actually been considered as thermoelectrics since they are known^25^, although the relative low atomic mass for the constitute elements and the simple crystal structure may lead to a high lattice thermal conductivity^25^,^26^,^27^.

As an important member among the elemental semiconductors, trigonal Te with the P3_1_21 space group undergoes a transition to a topological insulator phase^28^. However, it has been much less considered as a thermoelectric material.^29^,^30^,^31^ Available experimental results are limited to the electrical properties^29^,^30^,^31^,^32^ and low temperature thermal conductivity only^33^,^34^. Providing its intrinsically nested valence bands^30^,^32^,^35^,^36^, which are very similar to those of group IV and III–V semiconductors, as superior electronic performance can then be reasonably expected in tellurium. In addition, the relatively heavy atomic mass and the complexity in crystal structure in tellurium, as compared with the well-studied group IV and III–V semiconductors, should lead to a much lower lattice thermal conductivity^27^.

Guided by the concept of nested bands for high thermoelectric performance, this work focuses on the thermoelectric performance of polycrystalline tellurium, a constitute element commonly used for producing conventional thermoelectrics including PbTe and Bi_2_Te_3_. The thermoelectric figure of merit, *zT*, as high as 1.0, achieved in a material as simple as elemental tellurium, demonstrating the validity of the concept. The achieved *zT* of ∼1.0 here is actually the highest among the reported element-based thermoelectrics^37^ including those are heavily alloyed, such as SiGe (refs 38, 39) and BiSb (refs 40, 41, 42). Furthermore, the obtained high *zT* fills the gap of elemental thermoelectrics showing high *zT* in the temperature range of 300–700 K as shown in Fig. 1, revealing the importance of tellurium as a thermoelectric material when the application circumstance strictly disallows precipitation, segregation or volatilization.

## Results

### Band structure of tellurium

**Figure 1:**
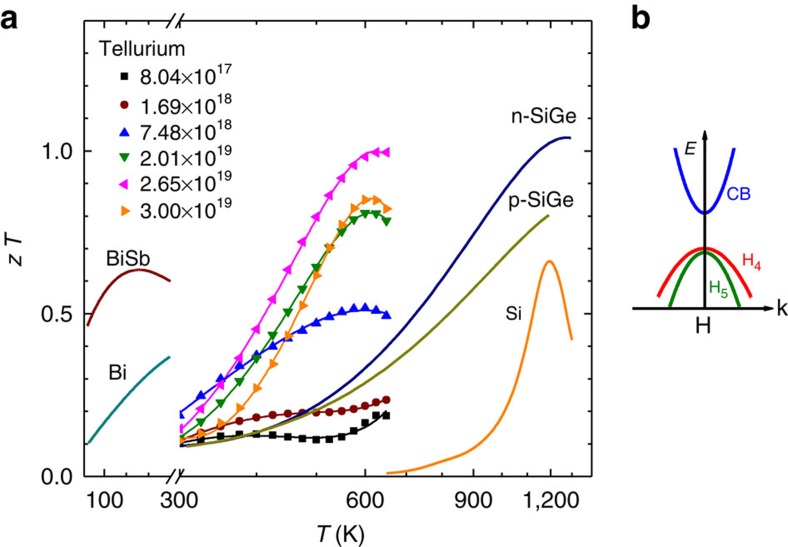
Survey of *zT* for elemental thermoelectrics. Temperature-dependent figure of merit (*zT*) for *p*-type ploycrystalline tellurium with different carrier concentrations shown in a unit of cm^−3^ (**a**). Both low temperature Bi/Bi–Sb alloys^40^ and high temperature Si (ref. 37)/Si–Ge alloys^39^ are included for comparison. *p*-type tellurium studied here shows a highest *zT* n the temperature range from 300 to 700 K, largely relies on its inherently nested valence bands (H4 and H5) as shown in **b**. The overlying lines in **a** are included to guide the eye.

It was reported^35^ as early as in 1950s, followed by a few other researchers in 1970s (refs 30, 32, 36, 43, 44, 45). Very recently, the detailed band structure has been given by theoretical calculations^28^,^46^. Regardless the different sources, the most important similarity, among the majority of the literatures, is that the nested valence bands at the H point in the Brillouin zone^30^,^32^,^36^,^43^,^44^,^45^,^46^ due to the spin-orbit coupling in tellurium. The four-orbital degenerated valence band at point H was split into two upper valence bands H4 and H5 and the lower doubly degenerate band H6 (refs 28, 46). Because the energy separation, either calculated^36^,^43^,^46^ (https://www.materialsproject.org/materials/mp-19/) or measured^47^, between the two upper valence bands (H4 and H5) is as small as 0.1 eV or less, they both contribute to the hole transport concurrently. On the other hand, the third valence band (H6) has a much lower energy^36^,^43^,^46^ and therefore is not influential to the electrical properties in the temperature and doping ranges studied here. Further due to the spin-orbital coupling, the first valence band, H4, may exhibit a weak camel's back in shape along the H→K direction^36^,^46^. However, this work focuses on the transport properties at temperatures >300 K, the resulting Fermi distribution broadening is significantly larger in energy than the difference between the extremums of the camel's back H4 band, leading to an unobservable effect on the electrical properties. Therefore, the band structure of Te can be approximated as the inset of Fig. 1 schematically, without including either the low energy H6 band or the camel's back feature for band H4.

It is then clear that the bands H4 and H5 are effectively nested in tellurium, giving a rise to the conducting channels for holes. This inherent band feature, and its effect on the thermoelectric performance is essentially similar with those caused by band convergence^11^,^22^,^48^, in which the converged bands unnecessarily have band extremum at the same k-space location. In this way, the two upper valence bands, with a valley degeneracy of 2 each, accumulate the hole pockets to a total number of 4 approximately, being comparable with 4∼6 that obtained in n-SiGe, Bi_2_Te_3_ and n-PbTe thermoelectrics.

### Carrier concentration-dependent transport properties

The transport properties were measured on single phased polycrystalline tellurium samples, where the trigonal structure and an average grain size of ∼100 μm are determined by X-ray diffraction and transmission electron microscope analyses (Supplementary Fig. 1). The transport properties in the directions along and perpendicular to the applied pressure of the hot press, are found to be nearly isotropic (Supplementary Fig. 2). Indeed, the experimental thermoelectric figure of merit, *zT*, of elemental tellurium is found to be as high as unity, being the highest among the reported element-based thermoelectrics including those are heavily alloyed such as SiGe (refs 38, 39) and BiSb (refs 40, 41, 42). This further leads to a fill-up to the gap of elemental thermoelectrics showing high *zT* in the temperature range from 300 to 700 K as shown in Fig. 1. The measured *zT* shows a good reproducibility (Supplementary Fig. 3) and comparability (Supplementary Fig. 4) to that measured by a different technique. The observed discrepancy on *zT* between the experimental results and the *ab initio* calculations^46^ is largely due to the difference on estimating the electronic thermal conductivity.

**Figure 2:**
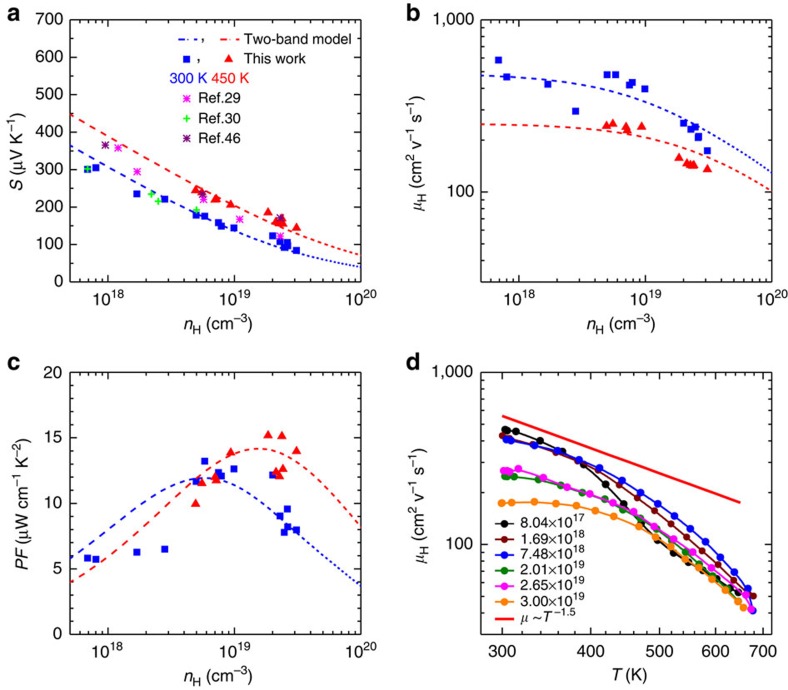
Transport properties for tellurium. Hall carrier concentration-dependent Seebeck coefficient (**a**) Hall mobility (**b**) and power factor (**c**) at 300 K (blue, green, purple and pink) and 450 K (red), and the temperature-dependent Hall mobility (**d**) with a comparison to literature results^29^,^30^,^46^. The dashed curves in (**a**–**c**) show the prediction based on a two-band Kane model with a scattering mechanism by acoustic phonons as evidenced from the temperature-dependent mobility (red line in **d**). Overlying lines in **d** are plotted to guide the eye and the carrier concentrations for the samples are shown in a unit of cm^−3^ (**d**).

In nested bands on the transport properties, the Hall carrier concentration dependence is given in Fig. 2. According to the above discussion on the band structure, a two-band (H4 and H5) model is used to understand the transport properties. Furthermore, due to the small band gap in tellurium, the band may be slightly nonparabolic and therefore needs to take into the first order of nonparabolicity (Kane band) into account^49^. The two Kane band model enables a reasonable prediction (dashed curves) on the hall carrier concentration-dependent Seebeck coefficient (Fig. 2a), Hall mobility (Fig. 2b) and power factor (Fig. 2c). The *ab initio* calculated^46^ Seebeck coefficient is slighter higher, which is presumably due to the fact that this method does not take into account the reduction of the band gap with increasing temperature^30^. As a result, the *ab initio* calculated^46^ power factor is also higher than the measurement (Supplementary Fig. 5). Similarly, this discrepancy can also be seen from the temperature-dependent transport properties as discussed below.

The Kane band model has shown similar success on understanding the transport properties of *n*-type PbTe (refs 19, 50, 51). It should be noted that the two-band model takes the band nonparabolicity, temperature-dependent effective mass and band gap^30^ into account. This model assumes a dominant charge carrier scattering by acoustic phonons, as evidenced in Fig. 2d by the observed temperature dependence of *T*^−1.5^ on the Hall mobility (*μ*_H_), because any other scattering mechanisms such as by grain boundaries, polar-optical phonons, and ionized impurities predict a dependence of *μ*_H_*∼T*^*p*^ with *p*≥−0.5. The even faster decrease in the Hall mobility than the *μ*_H_*∼T*^−1.5^ relationship at temperatures higher than 500 K, can be understood by the increased effective mass according to the two-band model.

**Figure 3:**
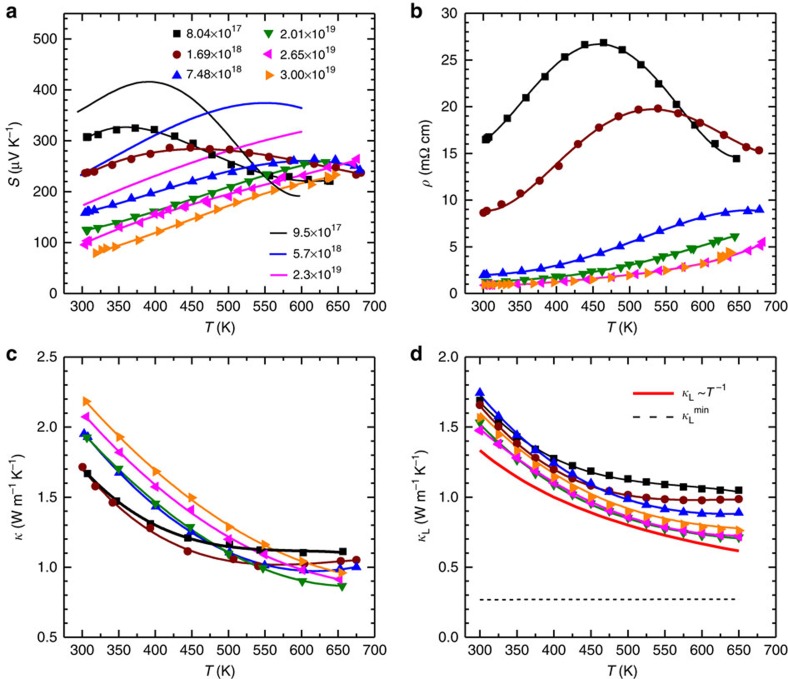
Transport properties as a function of temperature. Seebeck coefficient (**a**) resistivity (**b**) total thermal conductivity (**c**) and lattice thermal conductivity (**d**) for *p*-tellurium. The *ab initio* calculated Seebeck coefficient^46^ is included as solid curves for comparison in **a**. Majority of the samples studied here show a degenerate *p*-type semiconducting behaviour and a dominant phonon scattering by Umklapp process (red curve in **d**). The black dashed line in **d** shows the estimated minimal lattice thermal conductivity (*κ*_L_^min^) according to the Cahill model. Overlying lines are used to guide the eye and the carrier concentrations for the samples are shown in a unit of cm^−3^ in **a**.

This model further tells the optimal carrier concentration (*n*_opt_) that allows a maximum power factor to be achieved (Fig. 3c), and the resulting *n*_opt_ is found to be in the range of 1–3 × 10^19^ cm^−3^, depending the temperature and density of state effective mass^51^. The obtained *n*_opt_ is consistent with the *ab initio* calculation^46^, and is comparable with that of narrow band gap (*E*_g_<0.5 eV) thermoelectric semiconductors such as *n*-PbTe (ref. 51) and Bi_2_Te_3_ (ref. 52).

### Temperature-dependent transport properties

The temperature-dependent Seebeck coefficient (*S*) and resistivity (*ρ*) are shown in Fig. 3a and Fig. 3b, respectively. The doping effectiveness of arsenic can be seen from the significant decrease in both resistivity and Seebeck coefficient when the doping concentration increases. The positive sign of the Seebeck coefficient indicates the *p*-type conduction, which is consistent with our Hall coefficient measurements. Majority of the samples studied here show degenerated semiconducting behaviour, meaning a continuous increase in both resistivity and Seebeck coefficient with increasing temperature. The decrease in *ρ* and *S* at high temperatures can be ascribed to the existence of minority carriers, which is normally seen in narrow band gap semiconductors. The existence of minority carriers also lead to an increase in the high-temperature thermal conductivity, particularly in lightly doped samples.

The temperature-dependent total thermal conductivity and (*κ*) its lattice contribution (*κ*_L_) are shown in Fig. 3c and Fig. 3d, respectively. It can be seen from Fig. 3c that due to the effective doping by arsenic, the resulting reduced resistivity (Fig. 3b) leads to an increased electronic thermal conductivity (*κ*_E_) and therefore the total thermal conductivity. The electronic thermal conductivity can be determined by the Wiedeman–Franz law (*κ*_E_=*LT*/*ρ*), where the temperature-dependent Lorenz factor (*L*) is estimated by the two-Kane-band model. The lattice thermal conductivity is determined by subtracting the electronic contribution from the total thermal conductivity via (*κ*_L_=*κ*−*κ*_E_). The room temperature lattice thermal conductivity for unintentionally doped tellurium is ∼1.6 Wm^−1^ K^−1^, which is comparable with conventional thermoelectric lead chalcogenides^21^,^50^,^53^,^54^ and bismuth/antimony tellurides^52^,^55^, but significantly lower than those of group IV or group III–V semiconudctors^38^,^56^. The low lattice thermal conductivity is presumably due to the heavy atomic mass and relatively complex crystal structure^27^. Importantly, all the heavily doped samples show a nearly identical temperature dependence, and follow a nice *T*^−1^ decrease with increasing temperature, indicating a dominant phonon scattering by Umklapp process. According to the Cahill model^57^, the measured sound velocities (2,287 ms^−1^ for longitudinal and 1,410 ms^−1^ for transverse ones, respectively), enable a determination of minimal lattice thermal conductivity (*κ*_L_^min^), which is also shown in Fig. 3d. It can be seen that there should be a reasonably big room for a reduction on *κ*_L_ for an even higher *zT* through well-demonstrated strategies in thermoelectrics such as solid solution^38^,^58^ and nanostructuring^2^,^3^,^4^,^5^.

## Discussion

In summary, the inherently nested valence bands in tellurium enable an approximate hole pockets of 4, leading to a reasonably high power factor. In combination with its acceptably low thermal conductivity, elemental semiconducting tellurium surprisingly shows a high figure of merit, *zT*=1.0, and therefore nicely fills up the high performance gap from 300 to 700 K for elemental thermoelectrics. The guiding principle here introduces a pure electronic effect for discovering high thermoelectric performance materials, application of other well-demonstrated independent strategies such as alloying or nanostructruing for decreasing the lattice thermal conductivity, is expected to lead to an even higher *zT*.

## Methods

### Synthesis

Polycrystalline Te samples were prepared by melting high purity element (>99.99%) at 823 K for 8 h, followed by quenching in cold water and annealing at 673 K for 3 days. Dopants including phosphorus (P), arsenic (As), antimony (Sb) and bismuth (Bi) were used to tune the carrier concentration. It is found that As-doping is the most effective to achieve a high enough carrier concentration that is needed for realizing the high *zT* at high temperatures, and is therefore focused on in this study. The ingot materials were ground into fine powders and hot pressing^59^ at 673 K for 20 min under a uniaxial pressure of 90 MPa. The obtained dense pellet samples were a ∼12 mm in diameter and 1.5 mm in thickness.

### Structural characterization

The phase impurity was characterized by X-ray diffraction (Dandong Haoyuan Instrument Co. LTD). The samples for the transmission electron microscope observation were prepared by mechanical polishing, dimpling and ion milling with liquid nitrogen. STEM images were taken with a JEOL ARM 200 equipped with a probe corrector. The obtained pellet samples showed a density higher than 98% of the theoretical one, where microvoids with a size of microns can be occasionally observed.

### Transport property measurements

To be less involved in measurement uncertainties due to the possible hysteresis and the sample dimension determinations, the electrical transport properties including resistivity, Seebeck coefficient and Hall coefficient were simultaneously measured on the same pellet sample during both heating and cooling. The Seebeck coefficient was obtained from the slope of the thermopower versus temperature gradients of 0–5 K (ref. 60). The resistivity and Hall coefficient (*R*_H_) were measured using the van der Pauw technique under a reversible magnetic field of 1.5 T. For a comparison, the Seebeck coefficient and resistivity for two high performance samples were also measured using a ULVAC ZEM-3 system. The thermal diffusivity (*D*) was measured through laser flash method with the Netzsch LFA457 system. The heat capacity (*C*_p_) was assumed to be the Dulong–Petit limit and to be temperature independent, which is consistent with the literature result at room temperature^61^. The thermal conductivity was calculated via *к*=*d*C_p_*D*, where *d* is the density measured using the mass and geometric volume of the pellet. All the transport property measurements were performed under vacuum in the temperature range of 300–650 K. The sound velocity was measured using an ultrasonic pulse-receiver (Olympus-NDT) equipped with an oscilloscope (Keysight). The uncertainty for each measurement of transport property (including *S*, *σ* and *к*) is ∼5%.

## Additional information

**How to cite this article:** Lin, S. *et al*. Tellurium as a high performance elemental thermoelectric. *Nat. Commun.* 7:10287 doi: 10.1038/ncomms10287 (2016).

## Supplementary Material

Supplementary Information Supplementary Figures 1-5 and Supplementary Reference
