# Supplementary material for: Tellurium as a high-performance elemental thermoelectric
Source: Nat Commun. 2016 Jan 11;7:10287. doi: 10.1038/ncomms10287 (PMC4729895; doi:10.1038/ncomms10287)
Supplement: Supplementary Information — Supplementary Figures 1-5 and Supplementary Reference [file ncomms10287-s1.pdf]

Supplementary Figures

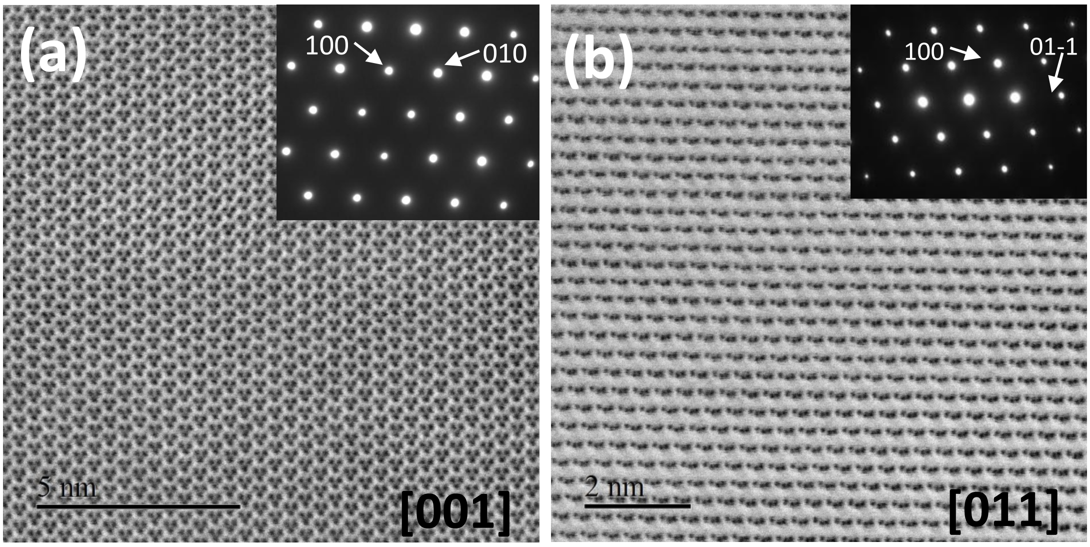

Supplementary Figure 1. Microstructure of tellurium. STEM images taken along [001] direction (a) and [011] direction (b) for the sample with the highest *zT*. Scale bar, 5 nm (a), 2 nm (b).

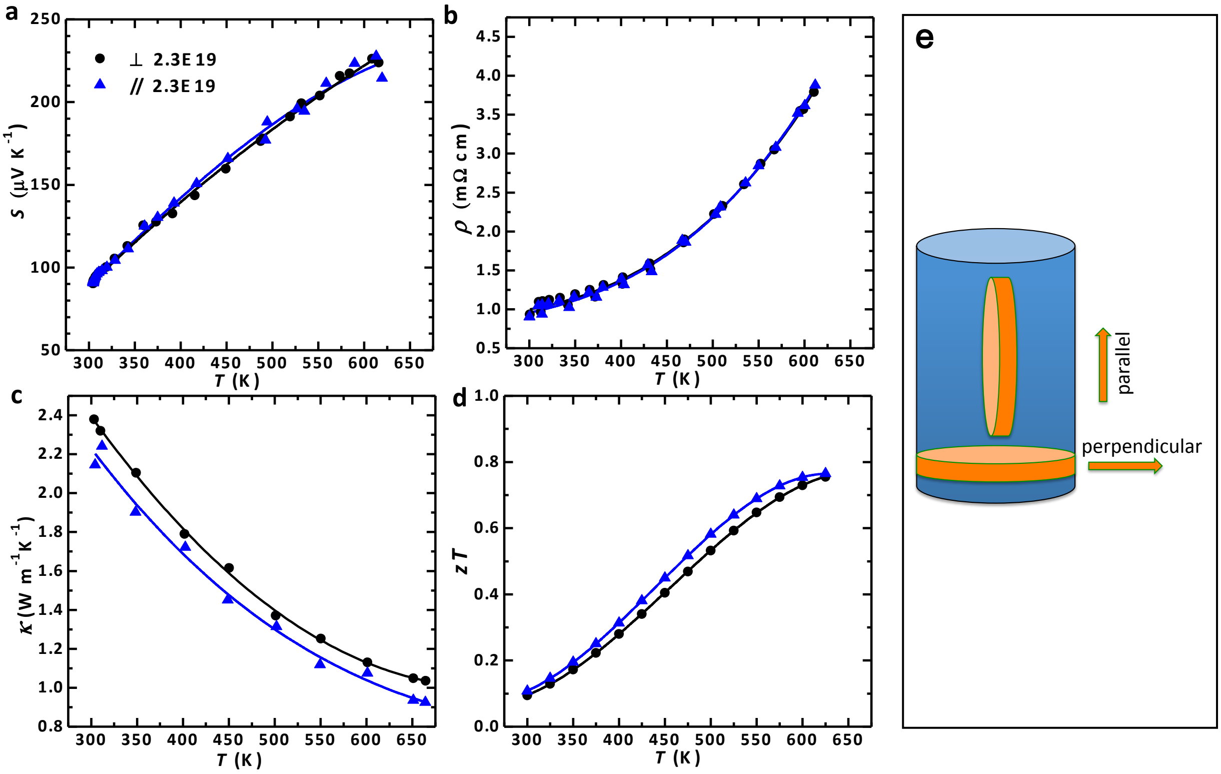

Supplementary Figure 2. Isotropic transport properties for tellurium. Temperature dependent Seebeck coefficient (a), resistivity (b), total thermal conductivity (c) and *zT* (d) in the directions parallel (blue) and perpendicular (black) to that of the pressure applied during the uniaxial hot press (e).

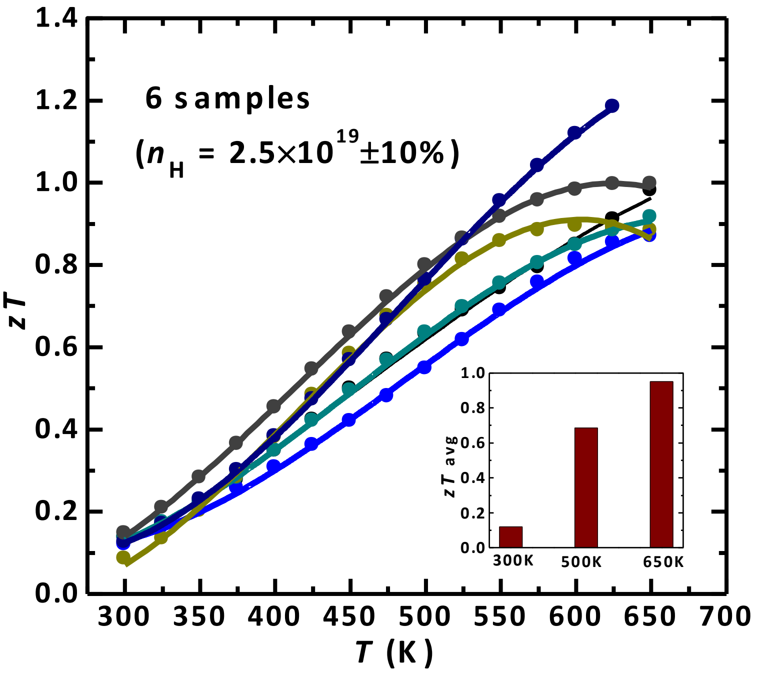

Supplementary Figure 3. Reproducibility of the thermoelectric performance. Temperature dependent zT for six samples with a carrier concentration of 2.5×10^{19} cm^{-3} ±10%. The peak zT ranges within of 0.8~1.2 and leads to an average value of 1.0 at 650K (inset).

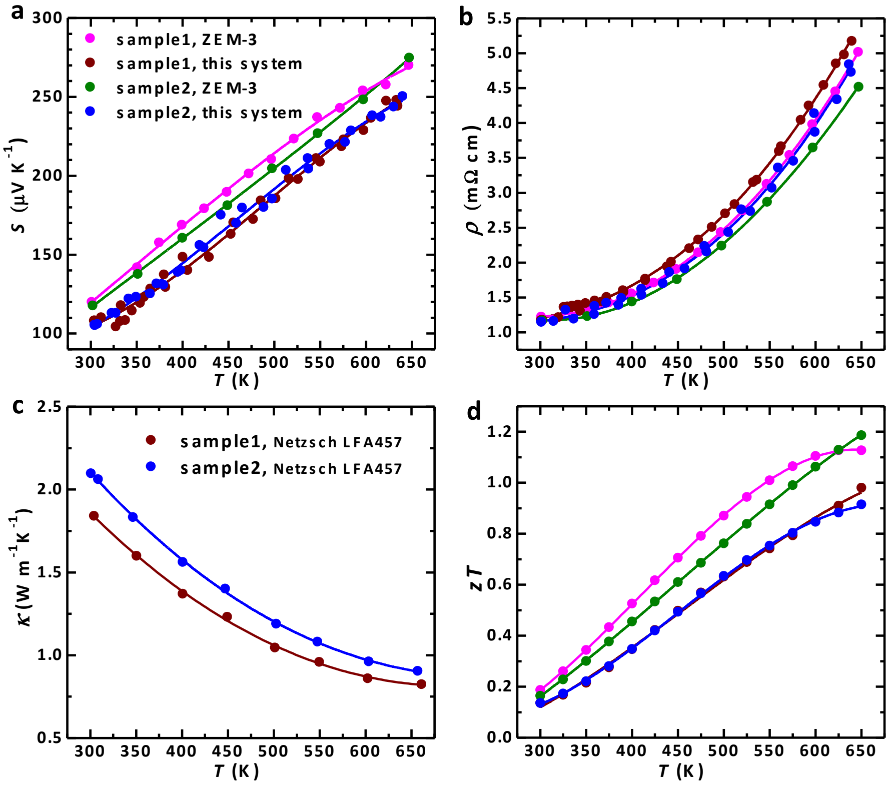

Supplementary Figure 4. Comparable measurements. Temperature dependent Seebeck coefficient (a), resistivity (b), total thermal conductivity (c) and *zT* (d) for two samples, measured by our system and ZEM-3.

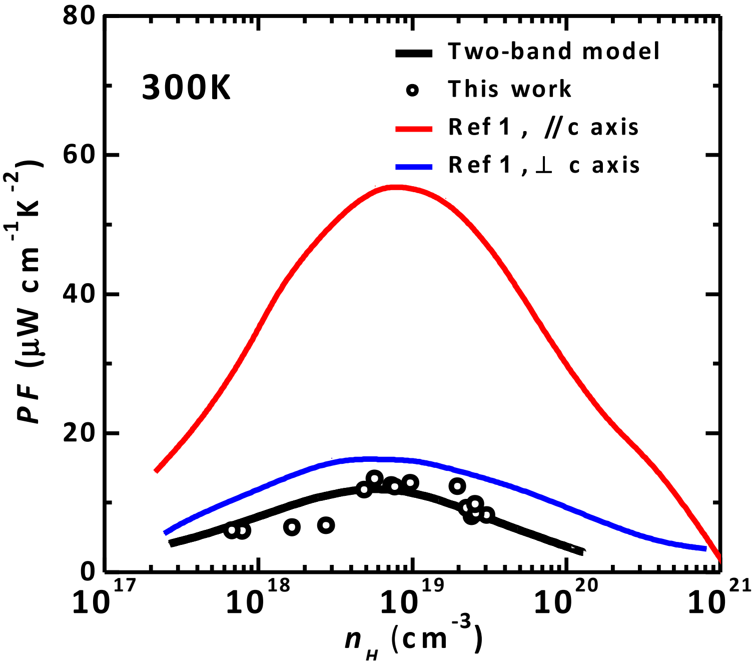

Supplementary Figure 5. Comparison between measured and calculated power factor. Power factor (*PF*) versus carrier concentration for tellurium. The blue and red curves are from the ab initio calculation[1] for directions along and perpendicular to the c axis, respectively. Our model prediction (curve) and measurement (circles) for polycrystalline tellurium are shown in black.

Supplementary Reference

1. Peng H, Kioussis N, Snyder GJ. Elemental tellurium as a chiral p-type thermoelectric material. *Phys Rev B* **89**, 195206 (2014).
